# Activation of the hypothalamic-pituitary-adrenal (HPA) axis contributes to the immunosuppression of mice infected with *Angiostrongylus cantonensis*

**DOI:** 10.1186/s12974-016-0743-z

**Published:** 2016-10-12

**Authors:** Ai-ling Chen, Xi Sun, Wei Wang, Jin-feng Liu, Xin Zeng, Jing-fan Qiu, Xin-jian Liu, Yong Wang

**Affiliations:** 1 Department of Pathogen Biology, Key Laboratory of Pathogen Biology of Jiangsu Province, Nanjing Medical University, 140 Hanzhong Road, Nanjing, Jiangsu 210029 China; 2 Wuxi Maternity and Child Health Hospital Affiliated to Nanjing Medical University, Wuxi, Jiangsu 214002 China; 3 Department of Parasitology, Zhongshan School of Medicine, Sun Yat-sen University, Guangzhou, Guangdong 510080 China

**Keywords:** *Angiostrongylus cantonensis*, Immunosuppression, Lymphopenia, Brain injury, Central nervous system, Hypothalamic-pituitary-adrenal axis

## Abstract

**Background:**

Immunosuppression has been described as a consequence of brain injury and infection by different mechanisms. *Angiostrongylus cantonensis* can cause injury to the central nervous system and eosinophilic meningitis to human. Both T cell and B cell immunity play an essential role in the resistance of the infection. However, whether brain injury caused by *A. cantonensis* infection can lead to immunosuppression is not clear. Therefore, the present study sought to observe the alteration of immune responses in mice infected with *A. cantonensis*.

**Methods:**

Mice were infected with 20 third-stage *A. cantonensis* larvae. The messenger RNA (mRNA) expression of inflammatory mediators in brain tissues was observed by qRT-PCR. Cell surface markers including CD3, CD4, CD8, CD19, B220, 7-AAD, annexin-V, IgM, AA4.1, and CD23 were evaluated by using flow cytometry. The immune functions of T and B lymphocytes were detected upon stimulation by ConA and antibody responses to a nonself antigen OVA, respectively. Activation of the hypothalamic-pituitary-adrenal axis was evaluated by analyzing the concentration of plasma corticosterone and levels of mRNA for corticotropin-releasing hormone, tyrosine hydroxylase, and c-fos.

**Results:**

*A. cantonensis* infection results in obvious immunosuppression evidenced as progressive spleen and thymus atrophy and significant decrease in the number of lymphocyte subsets including B cells, CD3^+^ T cells, CD4^+^ T cells, and CD8^+^ T cells, as well as reduced T cell proliferation at 21 days post-infection and antibody reaction to exogenous protein after infection. However, the sharp decrease of splenic and thymic cells was not due to cell apoptosis but to B cell genesis cessation and impairing thymocyte development. In addition, helminthicide treatment with albendazole on infected mice at 7 days post-infection could prevent immunosuppressive symptoms. Importantly, infected mice displayed hypothalamic-pituitary-adrenal axis activation, with peak responses occurring at 16 days post-infection, and glucocorticoid receptor antagonist could partially restore the infection-induced cessation of B cell genesis.

**Conclusions:**

Brain injury caused by *A. cantonensis* infection, like that of brain stroke and trauma, enhanced endogenous corticosteroid activity, resulting in peripheral immunosuppression.

**Electronic supplementary material:**

The online version of this article (doi:10.1186/s12974-016-0743-z) contains supplementary material, which is available to authorized users.

## Background

*Angiostrongylus cantonensis* (*A. cantonensis*) is a well-known food-borne causative agent of human meningoencephalitis in South-East Asia and many Pacific Islands. It is a result of human-associated spread from both its definitive host rats and intermediate hosts snails/slugs [1–4]. During the last decade, China and other countries reported several human angiostrongyliasis outbreaks, declaring it a public health problem [3, 5, 6]. Humans and mice are nonpermissive hosts of *A. cantonensis*. They become infected by eating the intermediate hosts or vegetables contaminated by the infective third-stage larvae. The infective larvae may invade intestinal tissues and migrate to brain, causing finally mechanical and inflammatory injuries to the central nervous system (CNS) [7–9].

After being infected with *A. cantonensis*, host immune responses played an important role in resisting infection and pathogenesis [10]. Depletion of CD4^+^ T cells by the monoclonal antibody impaired clearance of the worms, suggesting that CD4^+^ T cells play a protective role in *A. cantonensis* infection [11]. Peripheral blood mononuclear cells in patients with eosinophilic meningitis caused by *A. cantonensis* express a higher level of Th2 cytokines (e.g., IL-5) but lower levels of Th1 cytokines (e.g., IFN-γ and IL-2). However, treatment with albendazole inhibited IL-5, but increased the IL-2, IL-10, and IFN-γ gene expressions. These suggested that immune-pathology is mainly mediated by Th2 responses, and successful treatment changed the immune response from Th2 to Th1 dominance [12, 13].

Previously, studies on *A. cantonensis* largely focused on brain damage. However, the peripheral immune disorders caused by *A. cantonensis* infection are limited. Increasing evidence shows that the CNS and the immune system have bidirectional interaction [14]. CNS injury has profound effects on immune function. For example, patients after a stroke, traumatic brain injury, or spinal cord injury may have immune function defects including spleen and thymus atrophy, reduced peripheral blood lymphocyte counts, and impaired T cell activity [15–17]. This immunosuppression increases the risk of systemic infections such as pneumonia and urinary tract infections [14]. There was a case report showing that an *A. cantonensis* patient was complicated by secondary infections with methicillin-resistant *Staphylococcus aureus*, *Clostridium difficile*, and pneumonia. Yet the reason for secondary infections was not elucidated [18]. Numerous researches showed that brain injury may cause activation of hypothalamic-pituitary-adrenal (HPA) axis and induce glucocorticoid secretion [19–22]. Glucocorticoids could modulate the differentiation of B cells [23] and diminish B cell lymphopoiesis [24]. Glucocorticoids also induced thymic atrophy in mice infected with *Trypanosoma cruzi* [25] and *Mycobacterium avium* [26]. In addition, inhibiting HPA axis activation could prevent brain stroke-induced lymphocyte apoptosis, lymphopenia, and monocytic deactivation [16]. As for *A. cantonensis* infection, whether the CNS injuries could cause peripheral immune disorders is not clear.

In this study, we conducted both in vitro and in vivo studies to verify whether brain injury caused by *A. cantonensis* infection down-regulated the immune function. We found that *A. cantonensis*-infected mice showed significant signs of systemic immunosuppression. This was reflected in the decreased size of the spleen and thymus accompanied by lymphopenia, the impairment of cellular and humoral immune functions. Furthermore, the reduction of B and T cells was not caused by apoptosis but by the impairment of thymocyte development and the inhibition of B cell genesis following HPA axis activation.

## Methods

### Animals and infection

Female BALB/c mice aged 6–8 weeks were purchased from the Comparative Medicine Center of Yangzhou University (China) and maintained in the Animal Center of Nanjing Medical University according to guidelines approved by the Nanjing Medical University Animal Experiment and Care Committee (Approval No. 1403008). Mice were orally infected with 20 third-stage *A. cantonensis* larvae isolated from infected snails.

### qRT-PCR

Total RNAs were extracted from different tissues using RNA Isolation Reagent (Invitrogen, Carlsbad, CA) and reversely transcribed to produce cDNA (Fermentas, EU). Relative expression of messenger RNA (mRNA) species was determined by Real Time PCR with Faststart Universal SYBR Green PCR Master (Roche Diagnostics, USA) by ABI7300. The primer sequences were shown in Additional file 1: Table S1. Relative mRNA levels were normalized with β-actin, and results were expressed as fold amplification.

### Histopathological analysis of the brain and lungs

Mice were perfused transcardially with 0.9 % sodium chloride followed by 4 % paraformaldehyde (in 0.1 M phosphate buffer, PB, pH 7.4) after anesthetized with 2 % pentobarbital sodium. The lungs of the mice were harvested and fixed in 10 % formalin. After being embedded in paraffin, the brains and lungs were sliced into 4-μm-thick sections, stained with H&E, and examined by the microscope (ZEISS, Goettingen, Germany).

### Cell isolation

Heparinized tubes were used for blood collection. Single-cell suspensions from the thymus and spleen were prepared by forcing the tissues through a fine nylon mesh screen. The tibia and femur bones were used to prepare bone marrow cells. B and T cells were isolated from splenocytes by using magnetic beads following the manufacturer’s instructions (MACS, Miltenyi Biotech, Germany).

### Flow cytometry

Cell surface markers were stained with these specific antibodies: anti-CD19-PE, anti-CD3-APC, anti-CD4-PE, anti-CD8-FITC, 7-AAD, and anti-annexin-V-FITC (eBioscience); anti-CD19-APC, anti-B220-Per-cy5.5, anti-IgM-FITC, anti-AA4.1-PE, anti-CD23-eFluor647, and anti-CD8-APC (Biolegend).

For ex vivo analysis, cells were stimulated with 25 ng/mL PMA (Sigma-Aldrich) and 1 g/mL ionomycin (Sigma-Aldrich) in the presence of 0.66 μL/mL Golgistop (BD PharMingen) for 6 h at 37 °C, 5 % CO_2_. Intracellular staining of IFN-γ and IL-4 was performed using Transcription Factor Staining Buffer Set (eBioscience). Data was collected by FACS Calibur flow cytometer (BD Biosciences) and analyzed by FlowJo software (TreeStar, Ashland, OR).

For cell quantization, blood sample (100 μL) was stained with the specific antibodies: anti-CD19-FITC, anti-CD3-APC, anti-CD4-FITC, anti-CD8-FITC, anti-CD11c-APC, anti-F4⁄80-FITC, and anti-CD11b-APC. Erythrocytes were then lysed with Cal-lyse Lysing Solution (Invitrogen). After thoroughly mixing with 100 μL of Caltag Counting Beads (Invitrogen), 10,000 beads were acquired in the FACS Calibur flow cytometer for each sample.

### Proliferation assay

Splenic cells (2 × 10^5^/well) were seeded in 96-well flat-bottom tissue-culture plates and cultured for 72 h at 37 °C and 5 % CO_2_ with or without 2.0 μg/mL Concanavalin A (ConA) (Sigma-Aldrich). [^3^H] Thymidine (0.5 μCi, Amersham) was added at the last 18 h of the culture. Thymidine uptake was determined by liquid scintillation Counter (Beckman).

### Analysis of ex vivo cytokine production

Whole blood was diluted 1:5 in heparinized RPMI 1640 and incubated at 37 °C, 5 % CO_2_ [16]. For the analysis of TNF-α synthesis, samples were stimulated with 100 ng/mL LPS (Sigma-Aldrich) for 4 h. To analyze IFN-γ and IL-4 production, blood samples were stimulated with 100 μg/mL Con A for 24 h. Cultured supernatants were harvested for cytokine detection (Bender).

### Ovalbumin immunization

Naive and infected mice (7 dpi) were subcutaneously immunized with 100 mg of ovalbumin (OVA; Sigma-Aldrich) emulsified in an equal volume of TiterMax adjuvant (Sigma-Aldrich). After 14 days, the animals were sacrificed and sera were collected to assess OVA-specific antibodies using the ELISA method [27].

### Bacteriological analysis

The anesthetized mice were washed with 70 % ethanol. Blood was collected by cardiac puncture under sterile conditions. For the determination of CFU, 100 μL blood samples were serially diluted, plated onto blood agar plates, and incubated at 37 °C for 18 h.

### Western blot

Total proteins from B cells and T cells were extracted (Beyotime, China) and quantified using the BCA kit (Pierce, Rockford, IL). Lysates were separated on 12 % SDS–polyacrylamide gel electrophoresis and transferred to PVDF (Millipore, USA), followed by blocking in TBS/0.1 % Tween 20 with 5 % nonfat dry milk. Antibodies were used as following: rabbit anti-mouse Caspase 3 antibody (1:1000); goat anti-rabbit IgG HRP-conjugated antibody (1:2000); and mouse anti-β-actin antibody (1:1000) (Cell Signaling Technology).

### Albendazole treatment

Mice were infected with *A. cantonensis*, followed by intragastric administrations of albendazole (20 mg/kg/24 h) for seven consecutive days at 7 days post-infection (dpi).

### Corticosterone enzyme immunoassay

Blood samples were prepared at 8:00 am. Individual mice were bled within 30 s of being removed from their cage, which eliminates stress in cage-mates. Plasma was separated by using buffered citrate, and corticosterone levels were assessed by using the ELISA method (ENZO).

### Blockage of glucocorticoid receptors

RU486 (cayman) was dissolved in sesame oil at 10 mg/mL and administered (i.p, 50 mg/kg/24 h) at 10 dpi. The respective diluents were given to the control animals at the same time.

### Data analysis

Data was analyzed using two-tailed Student’s *t* test for the comparison between two groups. A *p* value <0.05 was considered statistically significant. All statistical analyses were operated by GraphPad Prism software 4.0 (GraphPad Software Inc., San Diego, CA).

## Results

### *A. cantonensis* infection-induced brain inflammation of mice

**Fig. 1:**
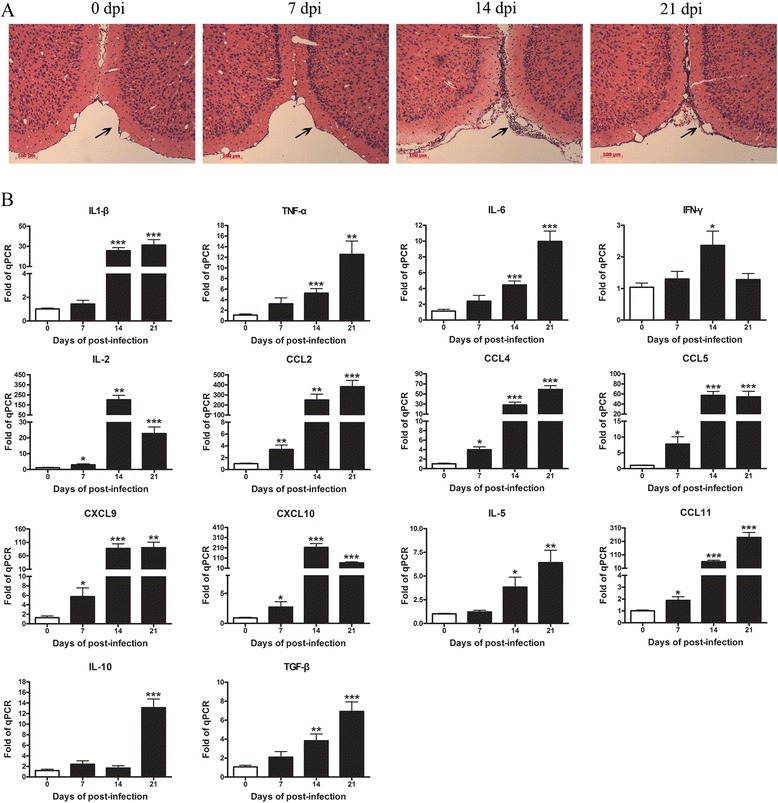
*A. cantonensis* infection of mice induced brain inflammation. Mice were orally infected with 20 third-stage larvae and sacrificed at 0, 7, 14, and 21 dpi, respectively. Brain tissues were harvested for **a** pathology evaluated by H&E staining. **b** Brain gene expressions of inflammatory cytokines were tested by RT-PCR. Each group contained five to six mice. One of two independent experiments with similar results is shown. Values were shown as mean ± SEM. One-way ANOVA was used for statistical analysis. *, results differed from the control group; *P < 0.05; **P < 0.01; ***P < 0.001

In this study, we orally infected mice with 20 third-stage larvae. Hemorrhages and tissue edema of the brain surface were observed macroscopically in *A. cantonensis*-infected mice at 21 dpi. Histological sections revealed that meninges thickened at 14 dpi, exhibiting severe meningitis by infiltrating a large number of inflammatory cells. However, at 21 dpi, the number of infiltrating inflammatory cells was reduced and moderate meningitis was observed (Fig. 1a). We also investigated the inflammatory mediators by determining mRNA expression of cytokines (e.g., IL-1α, TNF-α, IL-6, IFN-γ, and IL-2) and chemokines (e.g., CCL2, CCL4, CCL5, CCL11, CXCL9, and CXCL10) in the brain. At 14 and 21 dpi, we found generally high levels of expression of inflammatory cytokines and chemokines. In addition, at 21 dpi, the expression of anti-inflammatory cytokines IL-10 and TGF-β as neuroprotective factors was also increased (Fig. 1b). These results revealed that *A. cantonensis* infection caused obvious brain inflammation.

### *A. cantonensis* infection led to the atrophy of lymphoid organ and lymphopenia

**Fig. 2:**
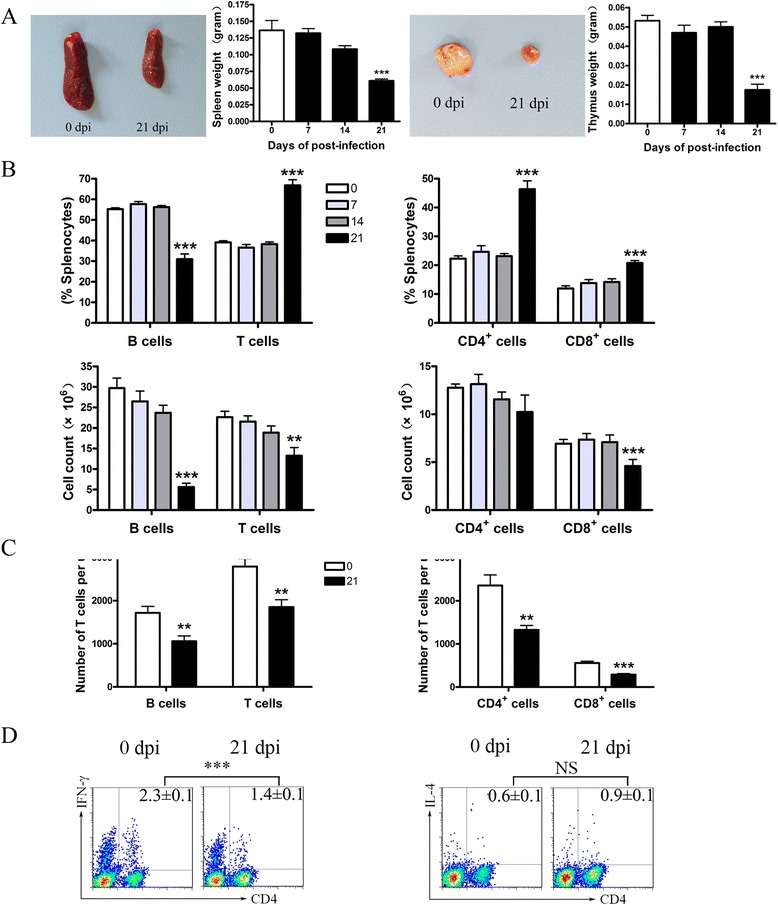
*A. cantonensis* infection led to lymphoid organ atrophy and lymphopenia of mice at 21 dpi. **a** Images of the spleen and thymus of control and 21 dpi mice. Spleen weight and thymus weight at different time-points of infection. **b** Percentages and absolute numbers of splenic B cells, T cells, CD4^*+*^ T, and CD8^*+*^ T cells. **c** The absolute number of total B cells, T cells, CD4^*+*^ T cells, and CD8^*+*^ T cells in peripheral blood. **d** Splenic cells from control and 21 dpi mice were gated on CD3^*+*^ T cells first, followed by the analysis for Th1, Th2, and T regulatory cells. Each group of mice comprised five to six animals. Data presented is one of three independent experiments. Values were shown as mean ± SEM. A one-way ANOVA is used to test the equality of three or more means by using variances. Two-tailed *t* test was used for two samples comparison. *, results differed from the control group; *P < 0.05; **P < 0.01; ***P < 0.001

To test whether brain injury caused by *A. cantonensis* infection would affect the peripheral immune system, we evaluated spleen and thymus morphology. At 21 dpi, spleens and thymuses showed serious atrophy (Fig. 2a).

To explore the underlying mechanisms of infection-caused thymus and spleen atrophy, we detected the cell subsets of spleen and peripheral blood by flow cytometry. As showed in Fig. 2b, there was a decrease in the percentage of B cells, but a significant increase in the percentage of T cells at 21 dpi compared with the controls. On further analysis of T lymphocyte subsets, the percentage of CD4^+^ and CD8^+^ T cells increased at 21 dpi compared with the controls. Furthermore, the absolute number of B cells in the spleen significantly decreased at 21 dpi. However, the absolute number of T cells, CD4^+^ T cells, and CD8^+^ T cells in the spleen only slightly declined compared to the control mice. Similar to the spleen, the absolute number of B cells, T cells, CD4^+^, and CD8^+^ T cells also decreased in white cell counts per milliliter of blood (Fig. 2c). Consistent with the previous study, a decrease in the proportion of Th1 cells was observed, while Th2 cells showed a slight increase at 21 dpi. These results suggested that the number of B cells and T cells was significantly affected by *A. cantonensis* infection.

### *A. cantonensis* infection led to the impairment of immune functions of mice

**Fig. 3:**
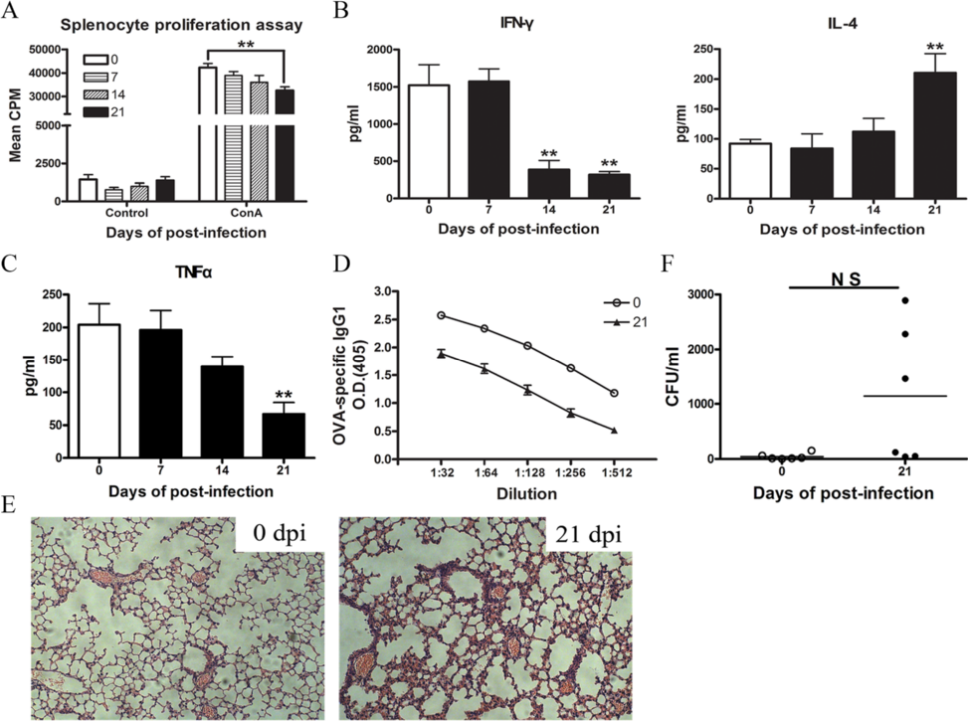
Cellular and humoral immune function of mice is impaired after *A. cantonensis* infection. **a** *A. cantonensis* infection dampened the proliferative capability of splenocytes. Splenocytes isolated from control and infected mice (7, 14, and 21 dpi) were stimulated with or without 2.0 mg/mL of ConA for 3 days, followed by evaluation of proliferation responses. **b**, **c** Blood samples were stimulated with ConA or LPS in vitro, and supernatants were collected for IFN-γ, IL-4, and TNF-α analysis by ELISA. **d** Data is expressed as dilution curves, with replicate samples plotted over multiple dilutions. All immunizations were done from control and 7 dpi mice, and antigen-specific antibodies were evaluated at 21 dpi to allow sufficient time for maximal T and B cell interactions and subsequent antibody synthesis. **e** Lungs from control and 21 dpi mice were collected for histological examination. **f** Blood samples from control and 21 dpi mice were collected for bacteriological analysis. Data is provided in CFU/mL of blood. Each group contained five to six mice. Values were shown as mean ± SEM. A two-tailed *t* test was used for statistical analysis. *, results differed from the control group; *P < 0.05; **P < 0.01; ***P < 0.001

To test whether *A. cantonensis* infection was also associated with immune function alteration, we detected the proliferative ability of T lymphocytes upon stimulation with ConA. T lymphocytes from mice at 21 dpi showed a significant decrease in proliferation compared to the controls (Fig. 3a). We further examined the ConA-induced IFN-γ and IL-4 production as well as the endotoxin-induced TNF-α secretion from blood as parameters of T lymphocyte and monocyte functions, respectively. We found reduced IFN-γ but increased IL-4 production at 21 dpi, resulting in a shift from Th1 to Th2 (Fig. 3b). Furthermore, endotoxin-induced TNF-α secretion showed a significant decrease at 21 dpi (Fig. 3c). Because of the importance of antibody synthesis to bacterial clearance and host defense [27], we tested antibody responses to a nonself antigen OVA. We found that the production of OVA-specific IgG1 in infected mice was dramatically reduced compared to the control which suggested that antibody synthesis was impaired in infected mice (Fig. 3d). This data indicated that infected mice exhibited impaired immune function. To further confirm this immunosuppression, we examined the histopathology of lungs and bacterial cultures from peripheral blood. At 21 dpi, histological examination of the lungs revealed typical signs of bacterial pneumonia (Fig. 3e). An increase in bacterial loads was also observed in blood at 21 dpi (Fig. 3f).

### Decline in the number of B and T cells was not caused by apoptosis

**Fig. 4:**
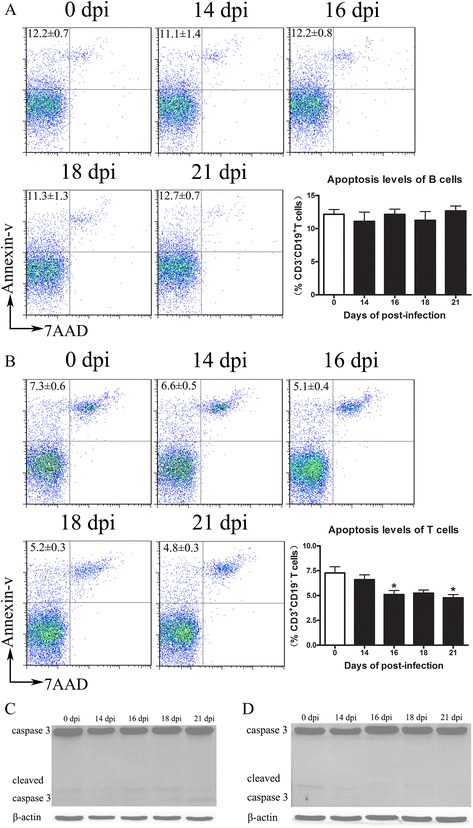
Decline in the number of B and T cells after *A. cantonensis* infection was not caused by cellular apoptosis. Splenic B (**a**) and T cells (**b**) were harvested from control and infected mice. Cellular apoptosis was examined by annexin-V/7-AAD staining. The expression of Caspase 3 from the purified B cells (**c**) and T cells (**d**) was detected by western blot. Each group contained five to nine mice. One of three independent experiments with similar results is shown. Values were shown as mean ± SEM. A one-way ANOVA is used for statistical analysis. *, results differed from the control group; *P < 0.05; **P < 0.01; ***P < 0.001

To determine whether lymphopenia was caused by cell apoptosis, we performed flow cytometric analysis. Compare with controls, B cells (Fig. 4a) and T cells (Fig. 4b) from infected mice showed no increase in apoptosis. Furthermore, we measured the levels of pro-caspase-3 and active capsase-3 from B cells (Fig. 4c) and T cells (Fig. 4d). Active caspase-3 was not detected in control and infected mice, indicating that the decrease of B and T cells was not due to cell apoptosis.

### *A. cantonensis* infection-induced inhibition of B cell genesis in the bone marrow (BM) and the impairment of thymocyte development of mice

**Fig. 5:**
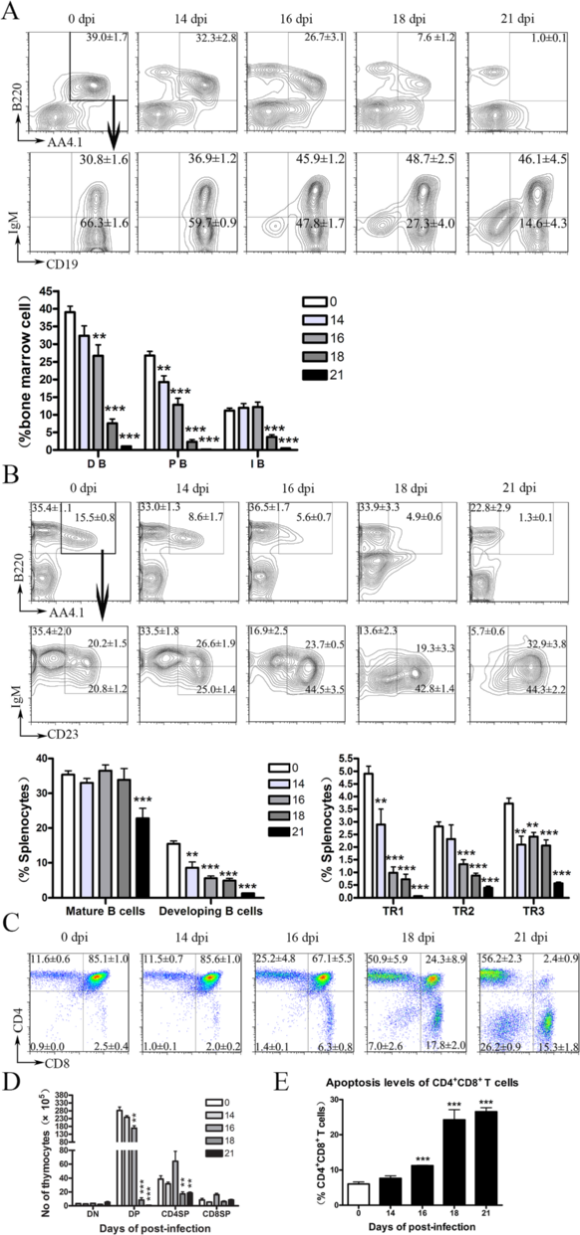
Inhibition of B cell genesis in the bone marrow and impairment of thymocyte development in *A. cantonensis*-infected mice. **a** Frequency of B cell progenitors in BM is reduced following infection. Developing B cells (DB) in the BM were gated on B220^+^AA4.1^+^ cells first (*upper panels*) and further analyzed for IgM^+^CD19^+^ immature (IB) and IgM^−^CD19^+^ pro-/pre-B cell (PB) populations (*lower right panels*). **b** Frequency of developing B cell in the spleen is reduced after infection. Mature B cells in the spleen were gated on B220^+^AA4.1^+^ cells. Developing B cells were gated on B220^+^AA4.1^+^ cells first (*upper panels*) and further analyzed for IgM^hi^CD23^−^ TR1, IgM^hi^CD23^+^ TR2, and IgM^lo^CD23^+^ TR3 developing B cells (*lower right panels*). **c** Proportion of thymocyte subsets in control and infected mice at 14, 16, 18, and 21 dpi. **d** Number of thymocyte subsets in control and infected mice at 14, 16, 18, and 21 dpi. **e** CD4^+^CD8^+^ T cells of thymus were investigated for apoptosis by annexin-V labeling. Each group contained four to six mice. One of three independent experiments with similar results is shown. Values were shown as mean ± SEM. A two-tailed *t* test was used for statistical analysis. *, results differed from the control group; *P < 0.05; **P < 0.01; ***P < 0.001

Following the pro-B and pre-B cell stages, developing B cells in the BM enter the immature B cell stage and migrate to the periphery as transitional (TR) B cells. Splenic TR cells further develop into TR1, TR2, and TR3 subsets based on differential CD23 and IgM expression levels [28]. Our results showed that *A. cantonensis* infection reduced splenic B cell numbers, which was not due to apoptosis. Such reductions may reflect decreased B cell genesis, a direct loss of all or some mature B cell subsets, or both. To confirm this hypothesis, we examined developmental B cell subsets in the BM (Fig. 5a) and developmental or mature B cell subsets in the spleen (Fig. 5b) during infection. At 21 dpi, among the developing B cell in BM, the proportions of pro/pre-B were significantly reduced and immature B cells were increased (Fig. 5a). Similarly, developing B cells in the spleen were also significantly reduced at 21 dpi (Fig. 5b). Among the developing B cells in the spleen, TR1 was profoundly reduced while TR2 and TR3 had increased at 21 dpi compared to control mice. The data suggested that B cell genesis ceased following infection with *A. cantonensis.*

*A. cantonensis* infection also led to a progressive shrinking of the thymus demonstrated by a reduction of mononuclear cells (Fig. 2a). Our data showed that after 16 dpi, infection led to the increase of relative proportions of CD4^−^CD8^−^, CD4^+^CD8^−^, and CD4^−^CD8^+^ populations. However, the proportion of CD4^+^CD8^+^ cells significantly decreased (Fig. 5c). On average, the number of CD4^+^CD8^+^ population underwent almost depletion at 21 dpi (Fig. 5d), which was due to apoptosis (Fig. 5e).

### Brain injury caused by *A. cantonensis* infection-induced lymphopenia and HPA axis activity of mice

**Fig. 6:**
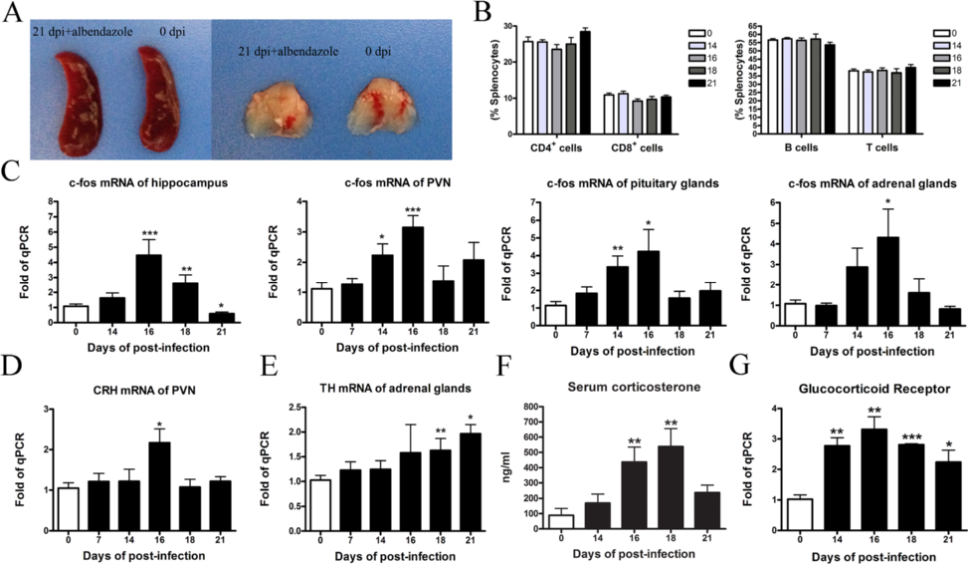
Brain injury caused by *A. cantonensis* infection-induced lymphopenia and HPA axis activity. **a** Mice were infected with *A. cantonensis* first, followed by the albendazole treatment from 7 to 14 dpi. Images of the spleen and thymus of 21 dpi mice were shown. Naïve mice were used as control. **b** Percentages of splenic B cells, T cells, CD4^+^ T, and CD8^+^ T cells in infected mice (14, 16, 18, and 21 dpi) with albendazole treatment. Naïve mice were used as control. **c** C-fos mRNA levels in the hippocampus, PVN, pituitary gland, and adrenal glands. **d** CRH mRNA levels in the PVN. **e** TH mRNA levels in the adrenal glands. **f** Serum corticosterone levels in infected mice (14, 16, 18, and 21 dpi) and control mice. **g** Glucocorticoid receptor mRNA levels in the BM from infected mice and control mice. Each group contained four to six mice. One of two independent experiments with similar results is shown. Values were shown as mean ± SEM. A two-tailed *t* test was used for statistical analysis. *, results differed from the control group; *P < 0.05; ***P < 0.001

To determine whether the decrease of lymphocytes was due to brain injury, we did helminthicide experiments. Mice at 7 dpi were treated with albendazole for seven consecutive days before the larvae were enriched in brain tissue. We observed that both the spleen and thymus showed no atrophy symptoms at 21 dpi (Fig. 6a). In addition, the subsets of lymphocyte, including B cells, T cells, CD4^+^, and CD8^+^ T cells, did not reduce after albendazole treatment (Fig. 6b). This data demonstrated that lymphopenia was likely caused by brain injury after infection.

As high levels of stress mediators were known to be immunosuppressive after brain injury, we questioned whether corticosteroids were necessary for infection-induced immunosuppression. Jakovcevski described the analysis of HPA activation [29]. In this study, levels of c-fos mRNA (Fig. 6c) in the hippocampus, paraventricular nucleus (PVN), pituitary gland, and adrenal gland were up-regulated at 14 dpi and significantly increased at 16 dpi compared to control mice. A comparable result of corticotrophin-releasing hormone (CRH) mRNA in the PVN was also detected (Fig. 6d). At 18 and 21 days after infection, mice showed higher tyrosine hydroxylase (TH) mRNA levels in the adrenal gland compared with control groups (Fig. 6e). Levels of plasma corticosterone in infected mice had increased at 16 and 18 dpi (Fig. 6f). Subsequently, serum corticosterone levels dropped to baseline at 21 dpi. We further examined mRNA expression of glucocorticoid receptor in the BM and found that its expression had increased more than twofold at 16 dpi compared to control mice (Fig. 6g). These results strongly indicated that *A. cantonensis* infection induced the activation of HPA axis.

### RU486 treatment partially reversed the ceases of B cell genesis in *A. cantonensis*-infected mice

**Fig. 7:**
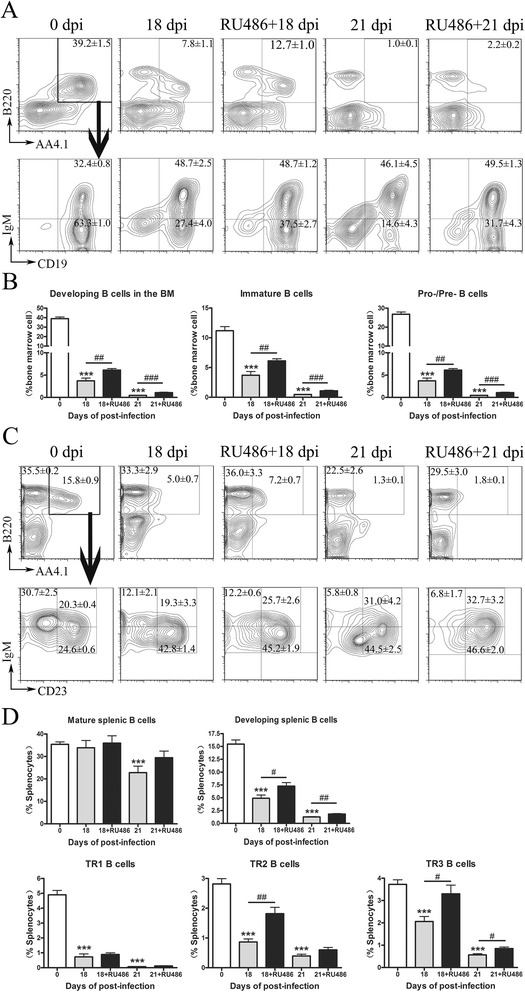
RU486 treatment partially reversed cessation of B cell genesis in *A. cantonensis*-infected mice. Mice were infected with *A. cantonensis* first and treated with RU486 or sesame oil daily since 10 dpi until sacrificed. **a**, **b** Developing B cells in the BM were gated on B220^+^AA4.1^+^ cells first (*upper panels*) and further analyzed for IgM^+^CD19^+^ immature (IMM) and IgM^−^CD19^+^ pro-/pre-B cell populations (*lower right panels*). **c**, **d** Frequency of developing B cell in the spleen is reduced after infection. Mature B cells in the spleen were gated on B220^+^AA4.1^+^ cells. Developing B cells were gated on B220^+^AA4.1^+^ cells first (*upper panels*) and further analyzed for IgM^hi^CD23^-^ TR1, IgMhiCD23^+^ TR2, and IgM^lo^CD23^+^ TR3 developing B cells (*lower right panels*). Each group contained four to six mice. One of two independent experiments with similar results is shown. Values were shown as mean ± SEM. A two-tailed *t* test was used for statistical analysis. *, results differed from the control group; *P < 0.05; **P < 0.01; ***P < 0.001. #, results differed from the infected and treated with sesame oil group; ^#^ *P* < 0.05; ^##^ *P* < 0.01; ^###^ *P* < 0.001

To assess the biological role of stress mediators in B cell genesis and thymocyte development, mice were infected with *A. cantonensis* and treated with RU486 (a steroid receptor type II antagonist) daily from 10 dpi. At 18 and 21 dpi, the number of BM pro/pre-B and immature B cells in RU486-treated mice was higher than that of control diluent-treated mice (Fig. 7a, b). Consistently, the number of splenic developing B cells in RU486 treated mice was also higher compared to the control diluent-treated group (Fig. 7c, d). However, RU486 treatment could not reverse the impairment of thymocyte development (Additional file 2: Figure S1). This data indicated that RU486 treatments partially reversed the cessation of B cell genesis in *A. cantonensis*-infected mice. It also supported the former results that *A. cantonensis* infection leads to HPA axis activation and the release of glucocorticoids associated with B cell output decline.

## Discussion

*A. cantonensis* causes severe neuropathological damages by invading and developing in brain tissue. Nonpermissive hosts (e.g., humans and mice) with *A. cantonensis* infection suffered more serious injuries and provoked more intense inflammatory responses compared to infected permissive hosts (e.g., rats) [10]. Brain injury not only damages brain tissues but also harms the peripheral immune system, resulting in severe systemic immunosuppression [30]. In addition, the secondary infection resulted from immunosuppression is a leading cause of death after brain injury [16, 31]. Taking this into consideration, we questioned whether the brain injury caused by *A. cantonensis* infection would influence immune system. We verified this hypothesis in the current study on *A. cantonensis* infection models of mice. Through the study, we showed the emergence of progressive atrophy of the thymus and spleen, as well as the reduced number and function of lymphocyte subsets. However, the decrease of lymphocytes was not attributable to cell apoptosis. Furthermore, we observed the activation of the hypothalamic-pituitary-adrenal axis that contacts central nervous system and immune system. We verified that the end products of HPA axis glucocorticoids promoted immune suppression.

In *A. cantonensis*-infected mice, larvae were first detected in cranial cavity at 10 dpi, and the highest number of larvae was found at 16 dpi. Damages including cavities and inflammation were found in the brain parenchyma by histological examination [32]. Consistently, we found that the number of infiltrating inflammatory cells increased at 14 dpi (Fig. 1a), while reducing at 21 dpi. Besides, the number of peripheral mononuclear cells had been sharply reduced at 21 dpi, leading to a reduction of cells entering the brain tissue. After we found immunosuppression at 21 dpi when the brain was damaged by parasites invasion, we questioned whether there was a relation between immunosuppression and brain injury. We designed a helminthicide experiment using albendazole from 7 to 14 dpi to deter the entry of larvae to the brain. We found that there were no immunosuppression symptoms including lymphopenia and lymphoid organ atrophy. This data suggested that the inhibition of immune responses was likely caused by parasite migration-based brain injury.

Previous studies showed that brain injury could lead to systemic down-regulation of innate and adaptive immunity. Prass demonstrated that cell apoptosis contributed to rapid and extensive loss of all lymphocyte subsets in lymphoid organs and peripheral blood after stroke [16]. However, our study showed no increase of apoptosis in B and T cells after *A. cantonensis* infection. Furthermore, the expression of chemokines (CCL2, CCL4, CCL5, CXCL9, CXCL10, and CCL11) and proinflammatory cytokines (IL-1, IL-6, and TNF-α) increased since 14 dpi. Chemokines could promote adhesion molecule expression by vascular endothelial cells. These cells further aggravate brain injury by allowing the infiltration of blood neutrophils, monocytes, macrophages, eosinophil, and T cells [33]. IL-1, IL-6, and TNF-α, which mainly come from activated microglial cells in the damaged brain, are commonly associated with HPA axis and sympathetic nervous system activation [19–22]. In this study, we observed that levels of mRNA for c-fos in the hippocampus, PVN, pituitary gland, and adrenal gland have increased at 14 dpi. Serum corticosterone concentrations were also markedly increased after 16 dpi. These results indicated that HPA axis activation might be associated to the decrease of lymphocytes after infection.

Stress hormones could both inhibit B lymphopoiesis and favor myelopoiesis, and they are elevated during acute injury [24, 27]. Subcutaneous implantation of corticosterone pellets into wild-type mice results in the alteration of B cell development in the bone marrow [34]. Reduction of B lymphopoiesis is associated with the increase of proinflammatory cytokine, such as TNF-α and IL-1, at the site of brain injury [35–37]. It has been speculated that both inflammatory cytokines and stress hormones impact B lymphopoiesis during injury [28]. These findings led us to ponder the role of corticosterone in inducing B cell development alteration after *A. cantonensis* infection. In this study, we observed that the proportion of B cell progenitors in BM had significantly decreased at 14 dpi compared to controls, and mature B cells in the spleen decreased at 21 dpi. Detailed research of developing B cell subsets in the spleen (TR1, TR2, and TR3) showed that TR1 was profoundly reduced, suggesting that B cell genesis ceases after *A. cantonensis* infection*.* Therefore, B cell genesis cessation contributes substantially to initial splenic B cell losses. Importantly, glucocorticoid receptor blocker RU486 administration to infected mice could partially reverse the alteration of B cell development in the bone marrow. Although the reconstitution of bone marrow B cell numbers is statistically significant, the magnitude of the increase is very small, and is likely to get limited major biological significance. However, these observations still demonstrated that corticosterone plays a role in the alteration of B cell development in *A. cantonensis* infection.

A common feature of variety of acute infections is severe atrophy of the thymus, largely reflecting intense lymphocyte depletion, particularly of cortical thymocytes which bear the CD4^+^CD8^+^ phenotype [38]. The thymic atrophy mechanism induced by *T. cruzi* or *M. avium* infection shows the increased thymocyte apoptosis due to glucocorticoid production [25, 39, 40]. In this study, we demonstrated that although the number of all cell populations decreased dramatically after infection, the most significant effect is on the immature CD4^+^CD8^+^ thymocyte population caused by apoptosis. When thymic atrophy and peripheral lymphopenia occur, the reconstitution of the immune system mainly depends upon the thymus to recover its ability to generate new T cells [41, 42]. To determine whether glucocorticoid increase would affect thymocyte numbers, we performed an in vivo functional assay using RU486. It was found that RU486 did not prevent thymocyte depletion following *A. cantonensis* infection*,* and the increase of apoptosis is just one of the mechanisms leading to thymic atrophy. The precise mechanisms responsible for thymic atrophy seen after *A. cantonensis* infection need further study.

The major clinical feature of *A.cantonensis* infection in human beings refers primarily to eosinophilic meningoencephalitis. Clinical symptoms appeared in patients on 7–35 dpi [43]. *A. cantonensis* infection of the human CNS could have devastating consequences. Severely affected patients may suffer from encephalitis, permanent neurologic injury, or even death [44]. Currently, albendazole is the drug of choice to treat *Angiostrongyliasis*. Dead worm lysis causes severe inflammatory response and can be expected to result in further damage of the CNS [45]. Therefore, patients under the treatment of high-dose corticosteroids would experience immune suppression. In addition, our data demonstrated that HPA axis activation contributes to the immunosuppression of infected mice, which especially increases the risk of bacterial infection in the lung. To our knowledge, this is the first report of an *A. cantonensis*-infected model with mediated immunosuppression. This model will be useful to further investigate the neuroimmunological mechanisms contributing to immunosuppression and to evaluate novel therapeutic approaches to prevent or reverse immunosuppression and its infectious complications.

## Conclusions

In summary, our study evidenced that the brain injury caused by *A. cantonensis* infection provides a powerful negative signal to the peripheral immune system. It ultimately induced a drastic state of immunosuppression and enhanced the risk of secondary infection. Our study suggested that clinicians should pay more attention to immunosuppression caused by *A. cantonensis* infection.

## Additional files

Additional file 1: Table S1. PCR primers used in this study. (DOC 41 kb)
Additional file 2: Figure S1. RU486 treatment did not reverse impairment of thymocyte development in *A. cantonensis*-infected mice. Mice were infected with *A. cantonensis* first and treated with RU486 daily since 10 dpi until sacrificed. Proportion of thymocyte subsets was detected. Each group contained 4–6 mice. Values were shown as mean ± SEM. A two-tailed *t* test was used for statistical analysis. *, results differed from the control group; ***P* < 0.01; ****P* < 0.001. (PDF 165 kb)

## Abbreviations

*A. cantonensis*: *Angiostrongylus cantonensis*
BM: Bone marrow
CNS: Central nervous system
CRH: Corticotrophin releasing hormone
DB: Developing B cells
HPA axis: Hypothalamic-pituitary-adrenal axis
IB: Immature B cells
IFN-γ: Interferon-gamma
PB: Pro-/pre-B cell
PMA: Phorbol myristate acetate
PVN: Paraventricular nucleus
SDS: Sodium dodecylsulfate
Th: Help T cell
TH: Tyrosine hydroxylase

## References

[1] Gelis S, Spratt DM, Raidal SR (2011). Neuroangiostrongyliasis and other parasites in tawny frogmouths (Podargus strigoides) in south-eastern Queensland. Aust Vet J.

[2] Maldonado A, Simoes RO, Oliveira AP, Motta EM, Fernandez MA, Pereira ZM (2010). First report of Angiostrongylus cantonensis (Nematoda: Metastrongylidae) in Achatina fulica (Mollusca: Gastropoda) from Southeast and South Brazil. Mem Inst Oswaldo Cruz.

[3] Wang J, Qi H, Diao Z, Zheng X, Li X, Ma S (2010). An outbreak of angiostrongyliasis cantonensis in Beijing. J Parasitol.

[4] Cowie RH (2013). Guest Editor’s message: eosinophilic meningitis caused by Angiostrongylus cantonensis, the rat lungworm: biology, distribution, epidemiology, detection, diagnosis, treatment, and management. Hawaii J Med Public Health.

[5] Lindo JF, Waugh C, Hall J, Cunningham-Myrie C, Ashley D, Eberhard ML (2002). Enzootic Angiostrongylus cantonensis in rats and snails after an outbreak of human eosinophilic meningitis, Jamaica. Emerg Infect Dis.

[6] Slom TJ, Cortese MM, Gerber SI, Jones RC, Holtz TH, Lopez AS (2002). An outbreak of eosinophilic meningitis caused by Angiostrongylus cantonensis in travelers returning from the Caribbean. N Engl J Med.

[7] Chen KM, Liu JY, Lai SC, Hsu LS, Lee HH (2006). Association of plasminogen activators and matrix metalloproteinase-9 proteolytic cascade with blood-CNS barrier damage of angiostrongyliasis. Int J Exp Pathol.

[8] Wang LC, Wan DP, Jung SM, Chen CC, Wong HF, Wan YL (2005). Magnetic resonance imaging findings in the brains of rabbits infected with Angiostrongylus cantonensis: a long-term investigation. J Parasitol.

[9] Jin E, Ma D, Liang Y, Ji A, Gan S (2005). MRI findings of eosinophilic myelomeningoencephalitis due to Angiostrongylus cantonensis. Clin Radiol.

[10] OuYang L, Wei J, Wu Z, Zeng X, Li Y, Jia Y (2012). Differences of larval development and pathological changes in permissive and nonpermissive rodent hosts for Angiostrongylus cantonensis infection. Parasitol Res.

[11] Lee JD, Wang JJ, Chang JH, Chung LY, Chen ER, Yen CM (1996). Role of T cell subpopulations in mice infected with Angiostrongylus cantonensis. J Helminthol.

[12] Diao Z, Chen X, Yin C, Wang J, Qi H, Ji A (2009). Angiostrongylus cantonensis: effect of combination therapy with albendazole and dexamethasone on Th cytokine gene expression in PBMC from patients with eosinophilic meningitis. Exp Parasitol.

[13] Du WY, Liao JW, Fan CK, Su KE (2003). Combined treatment with interleukin-12 and mebendazole lessens the severity of experimental eosinophilic meningitis caused by Angiostrongylus cantonensis in ICR mice. Infect Immun.

[14] Kamel H, Iadecola C (2012). Brain-immune interactions and ischemic stroke: clinical implications. Arch Neurol.

[15] Cruse JM, Lewis RE, Bishop GR, Kliesch WF, Gaitan E (1992). Neuroendocrine-immune interactions associated with loss and restoration of immune system function in spinal cord injury and stroke patients. Immunol Res.

[16] Prass K, Meisel C, Hoflich C, Braun J, Halle E, Wolf T (2003). Stroke-induced immunodeficiency promotes spontaneous bacterial infections and is mediated by sympathetic activation reversal by poststroke T helper cell type 1-like immunostimulation. J Exp Med.

[17] Wolk K, Docke WD, von Baehr V, Volk HD, Sabat R (2000). Impaired antigen presentation by human monocytes during endotoxin tolerance. Blood.

[18] Howe K (2013). A severe case of rat lungworm disease in Hawa’i. Hawaii J Med Public Health.

[19] Song C (2002). The effect of thymectomy and IL-1 on memory: implications for the relationship between immunity and depression. Brain Behav Immun.

[20] Jehn CF, Kuhnhardt D, Bartholomae A, Pfeiffer S, Schmid P, Possinger K (2010). Association of IL-6, hypothalamus-pituitary-adrenal axis function, and depression in patients with cancer. Integr Cancer Ther.

[21] Berthold-Losleben M, Himmerich H (2008). The TNF-alpha system: functional aspects in depression, narcolepsy and psychopharmacology. Curr Neuropharmacol.

[22] Woiciechowsky C, Schoning B, Daberkow N, Asche K, Lanksch WR, Docke WD (1999). Brain IL-1beta increases neutrophil and decreases lymphocyte counts through stimulation of neuroimmune pathways. Neurobiol Dis.

[23] Garvy BA, Telford WG, King LE, Fraker PJ (1993). Glucocorticoids and irradiation-induced apoptosis in normal murine bone marrow B-lineage lymphocytes as determined by flow cytometry. Immunology.

[24] Igarashi H, Medina KL, Yokota T, Rossi MI, Sakaguchi N, Comp PC (2005). Early lymphoid progenitors in mouse and man are highly sensitive to glucocorticoids. Int Immunol.

[25] Perez AR, Roggero E, Nicora A, Palazzi J, Besedovsky HO, Del Rey A (2007). Thymus atrophy during Trypanosoma cruzi infection is caused by an immuno-endocrine imbalance. Brain Behav Immun.

[26] Majlessi L, Simsova M, Jarvis Z, Brodin P, Rojas MJ, Bauche C (2006). An increase in antimycobacterial Th1-cell responses by prime-boost protocols of immunization does not enhance protection against tuberculosis. Infect Immun.

[27] Lucin KM, Sanders VM, Jones TB, Malarkey WB, Popovich PG (2007). Impaired antibody synthesis after spinal cord injury is level dependent and is due to sympathetic nervous system dysregulation. Exp Neurol.

[28] Oropallo MA, Held KS, Goenka R, Ahmad SA, O’Neill PJ, Steward O (2012). Chronic spinal cord injury impairs primary antibody responses but spares existing humoral immunity in mice. J Immunol.

[29] Jakovcevski M, Schachner M, Morellini F (2011). Susceptibility to the long-term anxiogenic effects of an acute stressor is mediated by the activation of the glucocorticoid receptors. Neuropharmacology.

[30] Offner H, Subramanian S, Parker SM, Wang C, Afentoulis ME, Lewis A (2006). Splenic atrophy in experimental stroke is accompanied by increased regulatory T cells and circulating macrophages. J Immunol.

[31] Vernino S, Brown RD, Sejvar JJ, Sicks JD, Petty GW, O’Fallon WM (2003). Cause-specific mortality after first cerebral infarction: a population-based study. Stroke.

[32] Guo PJ, Zhan XM, Gan M, Pan ZH, Yu YJ, Zhang MC (2008). Pathological change in the brain of mice infected with Angiostrongylus cantonensis. Zhongguo Ji Sheng Chong Xue Yu Ji Sheng Chong Bing Za Zhi.

[33] Barone FC, Feuerstein GZ (1999). Inflammatory mediators and stroke: new opportunities for novel therapeutics. J Cereb Blood Flow Metab.

[34] Laakko T, Fraker P (2002). Rapid changes in the lymphopoietic and granulopoietic compartments of the marrow caused by stress levels of corticosterone. Immunology.

[35] Ueda Y, Yang K, Foster SJ, Kondo M, Kelsoe G (2004). Inflammation controls B lymphopoiesis by regulating chemokine CXCL12 expression. J Exp Med.

[36] Dorshkind K (1988). IL-1 inhibits B cell differentiation in long term bone marrow cultures. J Immunol.

[37] Pineau I, Lacroix S (2007). Proinflammatory cytokine synthesis in the injured mouse spinal cord: multiphasic expression pattern and identification of the cell types involved. J Comp Neurol.

[38] Savino W (2006). The thymus is a common target organ in infectious diseases. PLoS Pathog.

[39] Roggero E, Perez AR, Tamae-Kakazu M, Piazzon I, Nepomnaschy I, Besedovsky HO (2006). Endogenous glucocorticoids cause thymus atrophy but are protective during acute Trypanosoma cruzi infection. J Endocrinol.

[40] Borges M, Barreira-Silva P, Florido M, Jordan MB, Correia-Neves M, Appelberg R (2012). Molecular and cellular mechanisms of Mycobacterium avium-induced thymic atrophy. J Immunol.

[41] Kalayjian RC, Spritzler J, Pu M, Landay A, Pollard RB, Stocker V (2005). Distinct mechanisms of T cell reconstitution can be identified by estimating thymic volume in adult HIV-1 disease. J Infect Dis.

[42] Smith KY, Valdez H, Landay A, Spritzler J, Kessler HA, Connick E (2000). Thymic size and lymphocyte restoration in patients with human immunodeficiency virus infection after 48 weeks of zidovudine, lamivudine, and ritonavir therapy. J Infect Dis.

[43] Wang QP, Lai DH, Zhu XQ, Chen XG, Lun ZR (2008). Human angiostrongyliasis. Lancet Infect Dis.

[44] Kwon E, Ferguson TM, Park SY, Manuzak A, Qvarnstrom Y, Morgan S (2013). A severe case of Angiostrongylus eosinophilic meningitis with encephalitis and neurologic sequelae in Hawa’i. Hawaii J Med Public Health.

[45] Wang J, Wei J, Zeng X, Liang JY, Wu F, Li ZY (2013). Efficacy of tribendimidine against Angiostrongylus cantonensis infection in the mice. Parasitol Res.

