# Supplementary material for: Activation of the hypothalamic-pituitary-adrenal (HPA) axis contributes to the immunosuppression of mice infected with Angiostrongylus cantonensis
Source: J Neuroinflammation. 2016 Oct 12;13:266. doi: 10.1186/s12974-016-0743-z (PMC5062856; doi:10.1186/s12974-016-0743-z)
Supplement: Additional file 1: Table S1. — PCR primers used in this study. (DOC 41 kb) [file 12974_2016_743_MOESM1_ESM.doc]

## Supplementary table 1. PCR primers used in this study

| mRNA | Forward Primer(5' -> 3') | Reverse Primer (5' -> 3') |
| --- | --- | --- |
| β-actin | GGCTGTATTCCCCTCCATCG | CCAGTTGGTAACAATGCCATGT |
| IL-1 β | GCAACTGTTCCTGAACTCAACT | ATCTTTTGGGGTCCGTCAACT |
| TNF-α | CCCTCACACTCAGATCATCTTCT | GCTACGACGTGGGCTACAG |
| IL-6 | TAGTCCTTCCTACCCCAATTTCC | TTGGTCCTTAGCCACTCCTTC |
| IFN-γ | ATGAACGCTACACACTGCATC | CCATCCTTTTGCCAGTTCCTC |
| IL-2 | GTGCTCCTTGTCAACAGCG | GGGGAGTTTCAGGTTCCTGTA |
| CCL2 | TTAAAAACCTGGATCGGAACCAA | GCATTAGCTTCAGATTTACGGGT |
| CCL4 | TTCCTGCTGTTTCTCTTACACCT | CTGTCTGCCTCTTTTGGTCAG |
| CCL5 | GCTGCTTTGCCTACCTCTCC | TCGAGTGACAAACACGACTGC |
| CCL11 | GAATCACCAACAACAGATGCAC | ATCCTGGACCCACTTCTTCTT |
| CXCL9 | GGAGTTCGAGGAACCCTAGTG | GGGATTTGTAGTGGATCGTGC |
| CXCL10 | CCAAGTGCTGCCGTCATTTTC | GGCTCGCAGGGATGATTTCAA |
| CCR2 | ATCCACGGCATACTATCAACATC | CAAGGCTCACCATCATCGTAG |
| CCR5 | TTTTCAAGGGTCAGTTCCGAC | GGAAGACCATCATGTTACCCAC |
| IL-10 | GCTCTTACTGACTGGCATGAG | CGCAGCTCTAGGAGCATGTG |
| TGF-β | CTCCCGTGGCTTCTAGTGC | GCCTTAGTTTGGACAGGATCTG |
| c-fos | CGGGTTTCAACGCCGACTA | TTGGCACTAGAGACGGACAGA |
| tyrosine hydroxylase | GTCTCAGAGCAGGATACCAAGC | CTCTCCTCGAATACCACAGCC |
| Corticotrophin releasing hormone | CCTCAGCCGGTTCTGATCC | GCGGAAAAAGTTAGCCGCAG |
| glucocorticoid  receptor | AGCTCCCCCTGGTAGAGAC | GGTGAAGACGCAGAAACCTTG |
