# Supplementary material for: Activation of the hypothalamic-pituitary-adrenal (HPA) axis contributes to the immunosuppression of mice infected with Angiostrongylus cantonensis
Source: J Neuroinflammation. 2016 Oct 12;13:266. doi: 10.1186/s12974-016-0743-z (PMC5062856; doi:10.1186/s12974-016-0743-z)

Supplementary Fig. 1 RU486 treatment did not reversed impairment of thymocyte development in *A.cantonensis* infection. Mice were infected with *A.cantonensis* first and treated with RU486 daily since 10 dpi until sacrificed. Proportion of thymocyte subsets was detected. Each group of mice comprised four to six animals. Values were shown as mean ± SEM. A Two-tailed T-test was used for statistical analysis. *, results differed from the control group; **, P＜0.01; ***, P＜0.001.

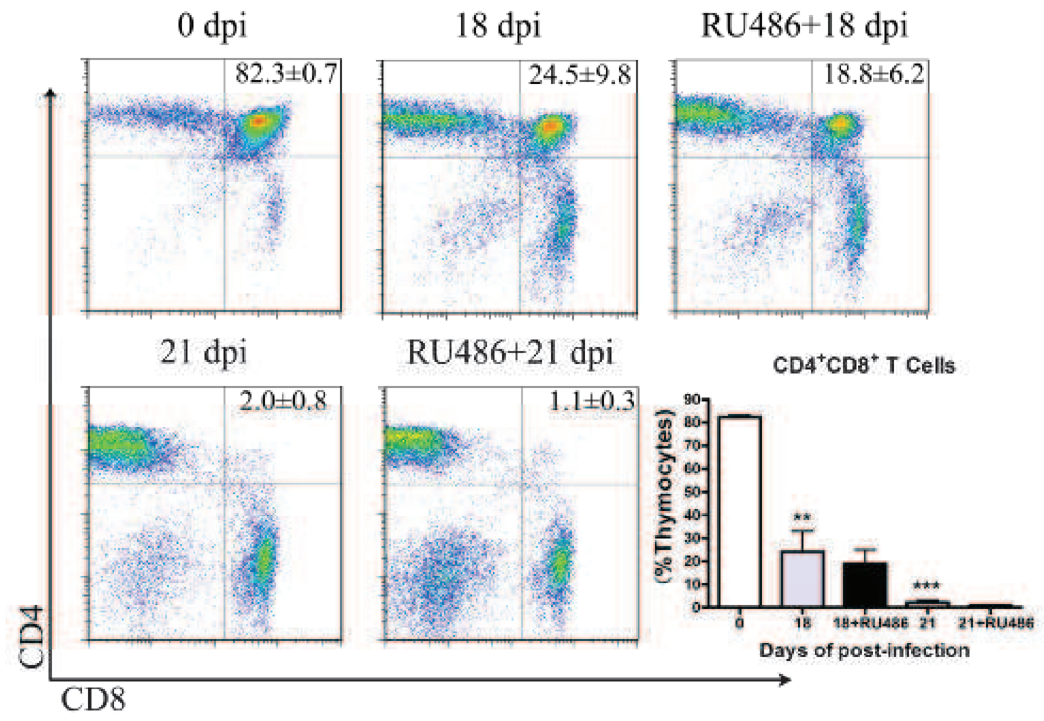

Supplement: Additional file 2: Figure S1. — RU486 treatment did not reverse impairment of thymocyte development in A. cantonensis-infected mice. Mice were infected with A. cantonensis first and treated with RU486 daily since 10 dpi until sacrificed. Proportion of thymocyte subsets was detected. Each group contained 4–6 mice. Values were shown as mean ± SEM. A two-tailed t test was used for statistical analysis. *, results differed from the control group; **P < 0.01; ***P < 0.001. (PDF 165 kb) [file 12974_2016_743_MOESM2_ESM.pdf]
